# Social Media Promotion of Raw Date Palm Sap and Emerging Nipah Virus Transmission Risk in Bangladesh: Descriptive Analysis of Multisource Data

**DOI:** 10.2196/84947

**Published:** 2026-04-02

**Authors:** Kamal Ibne Amin Chowdhury, Faruq Abdulla, S M Zafor Shafique, Laila Afroza, Nisharggo Niloy, James Soren, Amlan Chakma, Md. Maniruzzaman Howlader, Md Taizul Islam, Muhammad Rashedul Alam, Wasik Rahman Aquib, Dewan Imtiaz Rahman, Tonmoy Sarkar, Anika Farzin, Fateha Akther Ema, Shadman Sakib Choudhury, Mohammad Rezaul Karim, Sharmin Sultana, Mohammad Enayet Hossain, Md Sharful Islam Khan, Mohammed Ziaur Rahman, Trevor Shoemaker, Christina Spiropoulou, John D Klena, Tahmina Shirin, Sayera Banu, Joel M Montgomery, Syed Moinuddin Satter

**Affiliations:** 1 Infectious Disease, icddr,b, 68 Shaheed Tajuddin Ahmed Sarani, Mohakhali, Dhaka, 1212, Bangladesh, 880 1790665868; 2 Institute of Epidemiology, Disease Control and Research (IEDCR), Dhaka, Bangladesh; 3 Centers for Disease Control and Prevention (CDC), Atlanta, GA, United States

**Keywords:** Nipah virus, date palm sap, social media promotion, focused ethnography, infodemiology, zoonotic disease transmission, digital epidemiology

## Abstract

**Background:**

Nipah virus (NiV) infection is considered one of the deadliest infectious diseases, with a case fatality rate of approximately 71%. In Bangladesh, the primary risk factor for NiV infection is the consumption of raw date palm sap (DPS) contaminated with excreta from fruit bats (ie, members of the *Pteropodidae* family). Recently, the increasing use of social media among Bangladeshi youth has enabled business groups to widely advertise and sell raw DPS. This increased access, combined with young people perceiving consumption of raw DPS as an “adventurous event,” may facilitate an increase in incidences of NiV infection.

**Objective:**

We aimed to explore and document data from social media regarding raw DPS advertisements and consumption. Furthermore, we aimed to investigate the commercial distribution of raw DPS across the country.

**Methods:**

The data were accessed from common social media platforms used in Bangladesh, including Facebook and YouTube, between November 10, 2023, and January 31, 2024. We considered this period to capture public opinions, discussions, and reported incidents during the peak harvesting and consumption season of DPS. While DPS harvesting continues until March, early monitoring is essential for identifying the emerging concerns related to NiV transmission. Extracted variables were post dates and times, source locations, types of sources and posts, provider details (eg, sellers and *gachis*), post descriptions, user reactions, views, comments, and shares. Particular emphasis was placed on identifying the districts of both DPS suppliers and recipients. We used R version 4.3.2 and Stata version 15 for analyzing statistical data and QGIS for geographic data.

**Results:**

Of 556 social media posts, 361 (64.9%) were advertisements promoting raw DPS. Few DPS-related posts (n= 10, 1.8%) were associated with raising awareness about DPS consumption and NiV infection. In total, 473,724 people interacted with the social media posts. The identification of supplier and recipient districts revealed 14 source districts of raw DPS. The majority of raw DPS were distributed from Rajshahi, Naogaon, Rajbari, and Faridpur, which are among the most NiV-prone districts. During the data collection period, we observed an average of 996 (SD 377) liters of raw DPS sold per day from multiple *gachhis* (sap collectors) and sellers (vendors) in Rajshahi.

**Conclusions:**

The marketing of raw DPS through digital media platforms has increased customer interest, as evidenced by the notably high level of public engagement observed on this topic within the Bangladeshi social media context. Distributing raw DPS using digital platforms is a marketing tool that significantly increases the availability of raw DPS to previously hard-to-reach markets and potentially increases individuals’ exposure to NiV infection. This study recommends a multidisciplinary approach, which incorporates context-specific public health investigations, policymaking, and digital media surveillance to address emerging public health concerns.

## Introduction

Zoonotic diseases have emerged as a major public health concern in Bangladesh [1]. Among 1415 human pathogens identified globally, scientists reported that 61%, including Nipah virus (NiV), are zoonotic [2]. The World Health Organization has expressed growing concern about NiV by listing it as sixth of the next 10 possible pandemics, while Joi [3] highlighted NiV’s possibility of further mutation and rapid transmission. NiV is considered one of the most fatal zoonotic infectious diseases, causing severe health consequences, including neurological symptoms [4]. Bangladesh experiences human outbreaks of bat-borne NiV infection almost every year, with a case fatality rate of 71% [5]. In 2023, a total of 14 cases were reported, including 10 fatalities [6]. Therefore, notwithstanding the smaller number of infected individuals, NiV requires significant attention from researchers with multidisciplinary approaches.

*Pteropus* bats are the only known reservoir for NiV in Bangladesh, and contaminated date palm sap (DPS) with bat excreta is the route of transmission. DPS, considered a natural delicacy in Bangladesh, is a food source for bats in the winter season when other food sources are scarce. Local harvesters attach pots to date trees to collect raw sap. Bats often contaminate the sap by licking or urinating in the DPS collection pots while feeding from the pot at night [7]. Thus, the virus is transmitted through saliva and urine of the bats into DPS [8]. Laboratory experiments confirm that NiV can survive several days in raw DPS, and animals exposed to contaminated raw DPS exhibit NiV infection [9]. In addition, multiple studies conducted during outbreaks in Bangladesh demonstrated a higher likelihood of NiV-infected individuals having consumed raw DPS than control groups [10-13]. Therefore, the primary cause of NiV infection in Bangladesh is the consumption of contaminated raw DPS [14]. As evident from previous studies, the consumption of raw DPS is interrelated to human NiV infection [15]. Therefore, the increased consumption rate of raw DPS may contribute to the transmission of NiV. Additionally, the distribution of raw DPS from NiV-prone regions to other areas may increase the spread of NiV.

According to the Bangladesh Telecommunication Regulatory Commission, the number of internet users increased to 126 million in March 2023, up from 1.1 million in the early 2000s [16]. In Bangladesh, accelerated digitalization has led to the widespread adoption of social media platforms as an integral part of daily life [17]. Easy accessibility, along with several features for connecting with people, sharing daily life updates, entertainment, online business, and shopping, has made the use of Facebook (Meta Platforms, Inc), Instagram (Meta Platforms, Inc), Pinterest (Pinterest, Inc), Twitter (X Corp), and YouTube (Alphabet Inc) extremely popular among people, irrespective of age and gender, in Bangladesh [17]. The contemporary pattern of online shopping has reduced the difficulty of collecting products in person, playing a tremendous role in people’s lives. People can order any product from any part of the world, ranging from medicine to daily life essentials. As of 2022, a total of 50,000 small business pages run solely through Facebook for advertising and selling various products and food items within the country [18].

As the risk of contamination remains, the increased promotion and sales of raw DPS through digital media platforms can lead to a larger consumer base, facilitating the spread of NiV to new locations in Bangladesh. To evaluate this possibility, media monitoring surveillance could play a crucial role in documenting the data related to the potential social and behavioral activities and nuances contributing to NiV transmission through digital platforms. However, there is currently no evidence in the literature on how emerging digital marketplaces may facilitate or amplify exposure risks. To address this gap, our study aimed to measure the volume and nature of social media posts about raw DPS, map the networks of distribution facilitated by the digital platforms, and analyze public engagement with this content to evaluate the possibility of a new digitally driven risk of NiV transmission that may require novel surveillance and intervention strategies. In addition, we documented the volume of raw DPS and its daily distribution patterns from the original source to online sellers through on-site data collection in the Durgapur subdistrict of Rajshahi district.

## Methods

### Study Design and Setting

This study was a descriptive observational study that combined digital surveillance and on-site collection of data and was conducted between November 10, 2023, and January 31, 2024, in Bangladesh. The focus was on the promotion and distribution of raw DPS through social media platforms and its sales patterns in a Nipah-prone district.

### Data Collection

#### Online Data Collection

Data regarding the sale and promotion of raw DPS were systematically collected from commonly used social media platforms in Bangladesh, including Facebook, YouTube, LinkedIn (Microsoft Corp), Instagram, Twitter, and electronic media. The majority of relevant content was identified on Facebook, whereas negligible numbers of relevant posts were found from other platforms (YouTube, LinkedIn, Instagram, Twitter, and electronic media) and were therefore not included in the analysis. The data collection team consisted of a group of researchers from the International Centre for Diarrhoeal Disease Research, Bangladesh (icddr,b), representing multiple disciplines, including anthropology, sociology, epidemiology, and statistics. A systematic search was conducted across the platforms using commonly used keywords related to raw DPS, including *Bangla* terms such as “khejurer rosh” (*খেজুরের রস*), as well as English terms such as “date palm sap,” to identify relevant social media content. Duplicate and irrelevant posts were excluded. Only publicly available posts were included, and no direct interaction with users was undertaken. Because of privacy constraints, private inbox communications were inaccessible, and some data could not be captured (eg, recipient locations).

#### On-Site Data Collection

To determine the pattern of sales of raw DPS, an exploratory study was carried out in the Durgapur subdistrict of Rajshahi district, one of the Nipah-prone areas. Daily sales volume data (in liters) were collected through interviews with multiple *gachhis* (sap collectors) and sellers (vendors) and, when available, by sales records or logs. Information about districts receiving DPS shipments was also gathered during these interviews.

### Sampling and Inclusion Criteria

Social media posts included in the study were selected through a keyword-based systematic search during the study period. Posts were screened for relevance to raw DPS sales or promotion. The on-site sample consisted of sellers and *gachhis* who were available and consented to participate within the specified subdistrict during the data collection period.

### Variables

Extracted variables included the following: (1) date and time of post, (2) source location (if disclosed; no attempt was made to obtain undisclosed information), (3) provider details (name of seller or source, if publicly available), (4) post description and content, (5) user interaction indicators (reactions, views, comments, and shares), and (6) location of suppliers and recipients at the district level (if publicly disclosed).

### Handling of Missing Data

Any information that was not publicly available or accessible (eg, the location of recipients in private messages) was recorded as missing. No imputation was performed. Analyses were conducted on available data only.

### Addressing Bias and Limitations in Methods

The inclusion of publicly available posts and the use of a keyword-based search strategy could result in potential selection bias because it may not identify all the relevant content. The on-site data collection was limited to 1 subdistrict, which may not represent sales patterns in other regions. In the study design and interpretation, these limitations were considered.

### Sample Size Justification

As this was an exploratory and descriptive study, no formal calculation of the sample size was performed. The study period and the search keyword were designed to capture a comprehensive dataset reflecting the DPS promotion and sales during the peak season.

### Data Analysis

The data were summarized using descriptive statistics in R (version 4.3.2; R Foundation for Statistical Computing) and Stata (version 15; StataCorp). We used some graphical techniques, including epidemic curves and line-bar charts, to examine the temporal patterns. Quantum geographical information system was used to generate GPS-based maps.

### Operational Definitions

The following operational definitions were applied:

- Nipah-prone districts—districts where historical NiV outbreaks have occurred
- Non–Nipah-prone districts—districts where historical NiV outbreaks have not occurred
- Advertisement posts—posts that involve direct promotion of the sale of raw DPS, including posts offering price, quantity, delivery options, or seller contact information
- Promotional posts—posts encouraging consumption or highlighting perceived advantages (eg, fresh and healthy sap is available); however, no direct sales are made
- Awareness posts—posts warning about health risks or disease transmission or recommending avoidance of raw DPS

### Ethical Considerations

This study analyzed publicly accessible social media posts without interacting with the users. Following the standard digital epidemiology guidelines, all user identifiers, including names, profile images, and URLs, were removed before analysis to ensure privacy and confidentiality. No attempts were made to contact or identify social media users whose posts were included in the dataset. For on-site data collection, informed written consent was obtained from raw DPS sellers before collecting any information. Participation was voluntary, and no financial compensation was provided to participants. The research protocol (PR-22102) was reviewed and approved by the Research Review Committee and the Ethical Review Committee of icddr,b.

## Results

**Figure 1.**
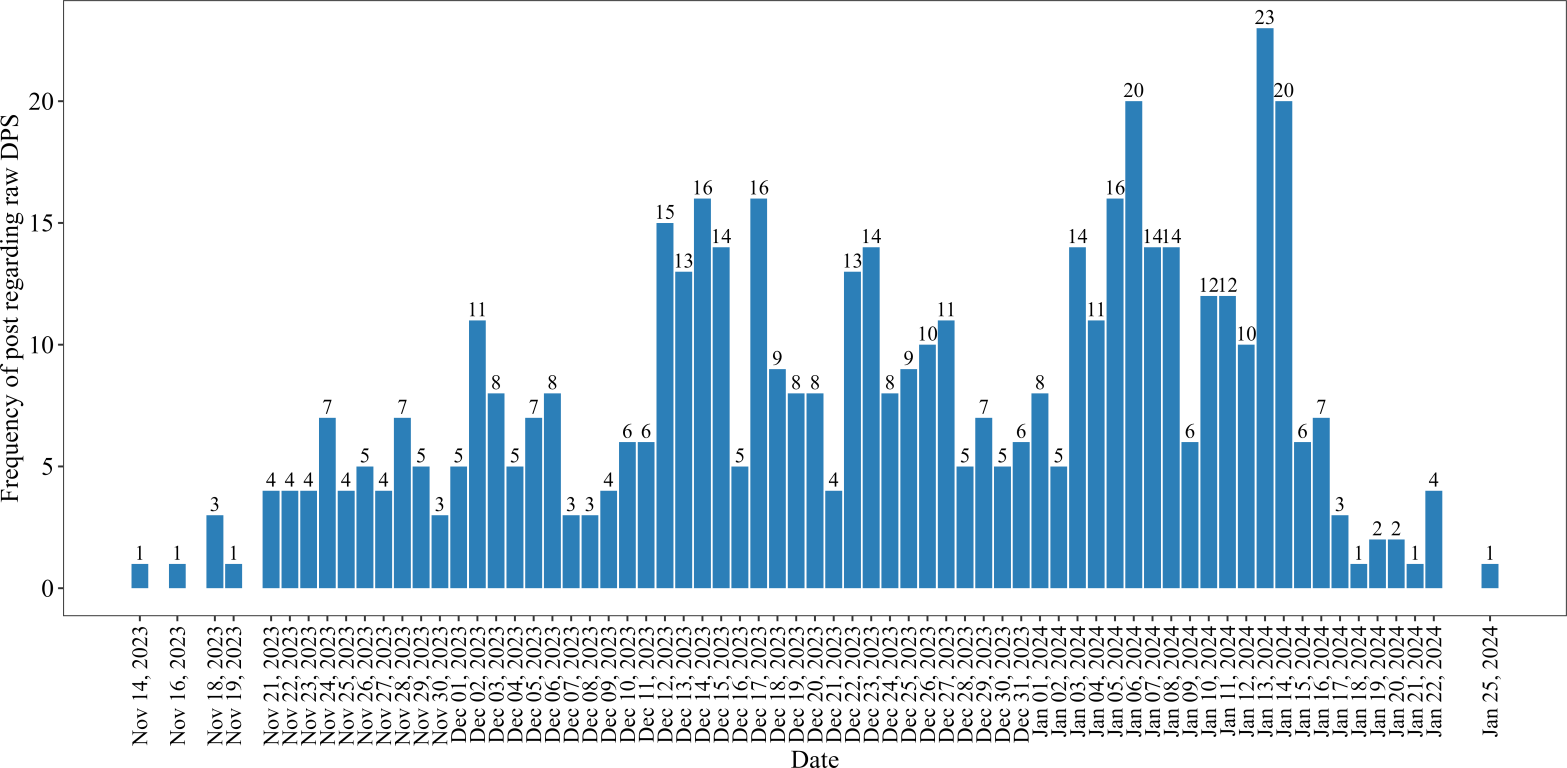
Epidemic curve of posts regarding raw date palm sap (DPS) on the selected social media platforms during the period from November 10, 2023, to January 31, 2024.

The frequency of posts related to the sale of raw DPS through digital media exhibited a dynamic pattern over the specified period (Figure 1). In particular, the most frequent postings were made by digital marketers between December 12, 2023, and January 14, 2024. The data demonstrate fluctuations in the number of posts, with the highest numbers observed on specific dates, such as December 14, 2023; December 17, 2023; January 6, 2024; January 13, 2024; and January 14, 2024, when 16 to 23 posts were posted per day.

Findings revealed various types of posts used to promote and engage with the audience through digital platforms (Table 1) for the marketing of raw DPS. Among these social media posts, we observed a large number of advertisements of raw DPS and found it to be approximately 64.9% (361/556) of the total posts. Additionally, promotional posts (approximately 170/556, 30.6%) highlighted different aspects of raw DPS, such as taste. In addition, we found some awareness posts regarding the risk of NiV and the consumption of raw DPS. Such posts were scarce in number, accounting for 1.8% (10/556) of posts.

**Table 1.** Types of posts regarding raw date palm sap (DPS) on selected social media platforms during the period from November 10, 2023, to January 31, 2024 (N=556).

Types of posts	Values, n (%)
Advertisements	361 (64.9)
Promotional posts	170 (30.6)
Awareness posts	10 (1.8)
Personal posts	6 (1.1)
News reports on DPS collection	2 (0.4)
Posts exploring DPS sales points	7 (1.2)

**Figure 2.**
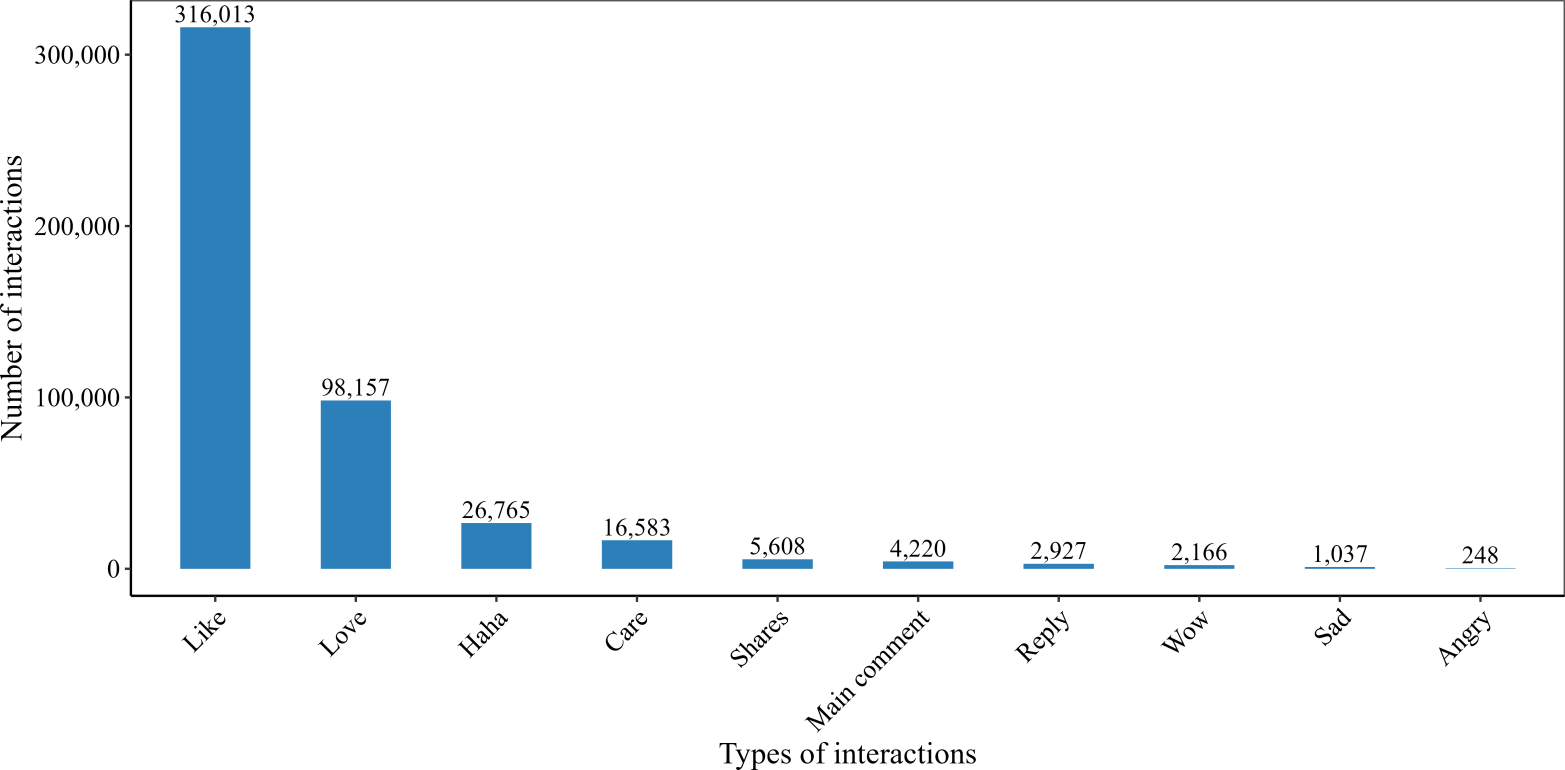
Interaction of people with posts regarding raw date palm sap on the selected social media platforms during the period from November 10, 2023, to January 31, 2024.

We analyzed human interaction with the posts regarding raw DPS, and the results are illustrated in Figure 2. We observed a total of 473,724 people interacting with posts regarding raw DPS through social media platforms such as Facebook. Among these interactions, a total of 459,684 (97%) people expressed positive reactions such as like, love, care, haha, and wow to the posts; however, only 1285 (0.3%) people expressed negative reactions such as sad and angry. Moreover, it is worth mentioning that 5608 (1.2%) people recirculated these posts by sharing them on their own or other people’s profiles.

**Figure 3.**
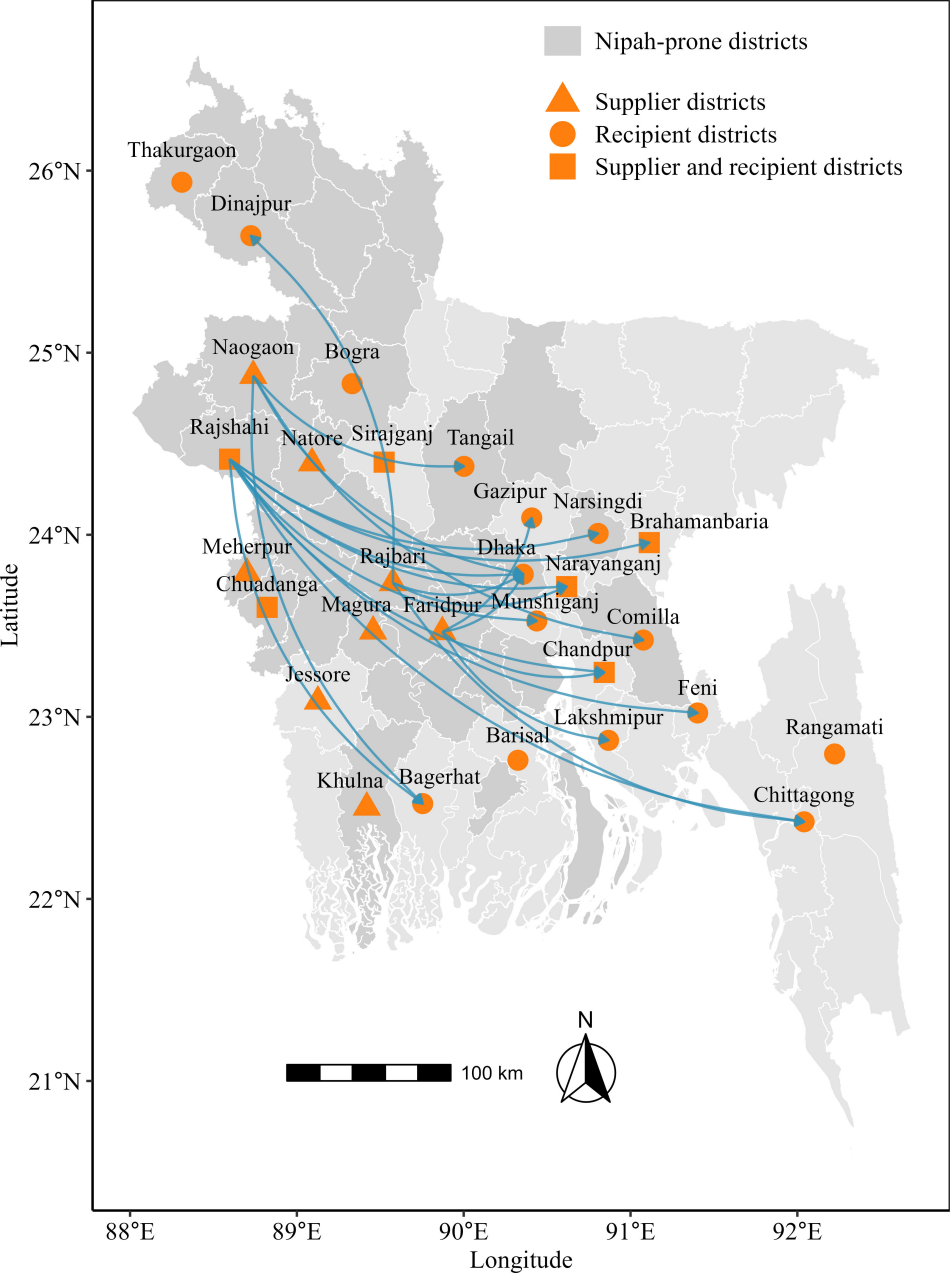
Geographic distribution of supplier and recipient districts and the distribution network of raw date palm sap in Bangladesh.

The identification of supplier and recipient districts shows the distribution network of raw DPS, as illustrated in Figure 3. Raw DPS was reportedly sourced from 14 districts and subsequently distributed to consumers across Bangladesh through digital platforms. Conversely, people in about one-third of the districts in Bangladesh collected raw DPS through digital platforms from the mentioned supplier districts. It is important to note that most of the raw DPS receiver districts were non–Nipah-prone areas of Bangladesh.

We examined the distribution pattern of raw DPS from 4 Nipah-prone districts (Rajshahi, Naogaon, Rajbari, and Faridpur) to other districts (Figure 3). We observed that the raw DPS from the districts of Rajshahi, Naogaon, Rajbari, and Faridpur was distributed to 9, 5, 4, and 4 districts, respectively. Raw DPS was supplied to some districts from multiple Nipah-prone districts. Notably, raw DPS from all the Nipah-prone districts is distributed to the Dhaka district, where the capital city of the country is located.

**Figure 4.**
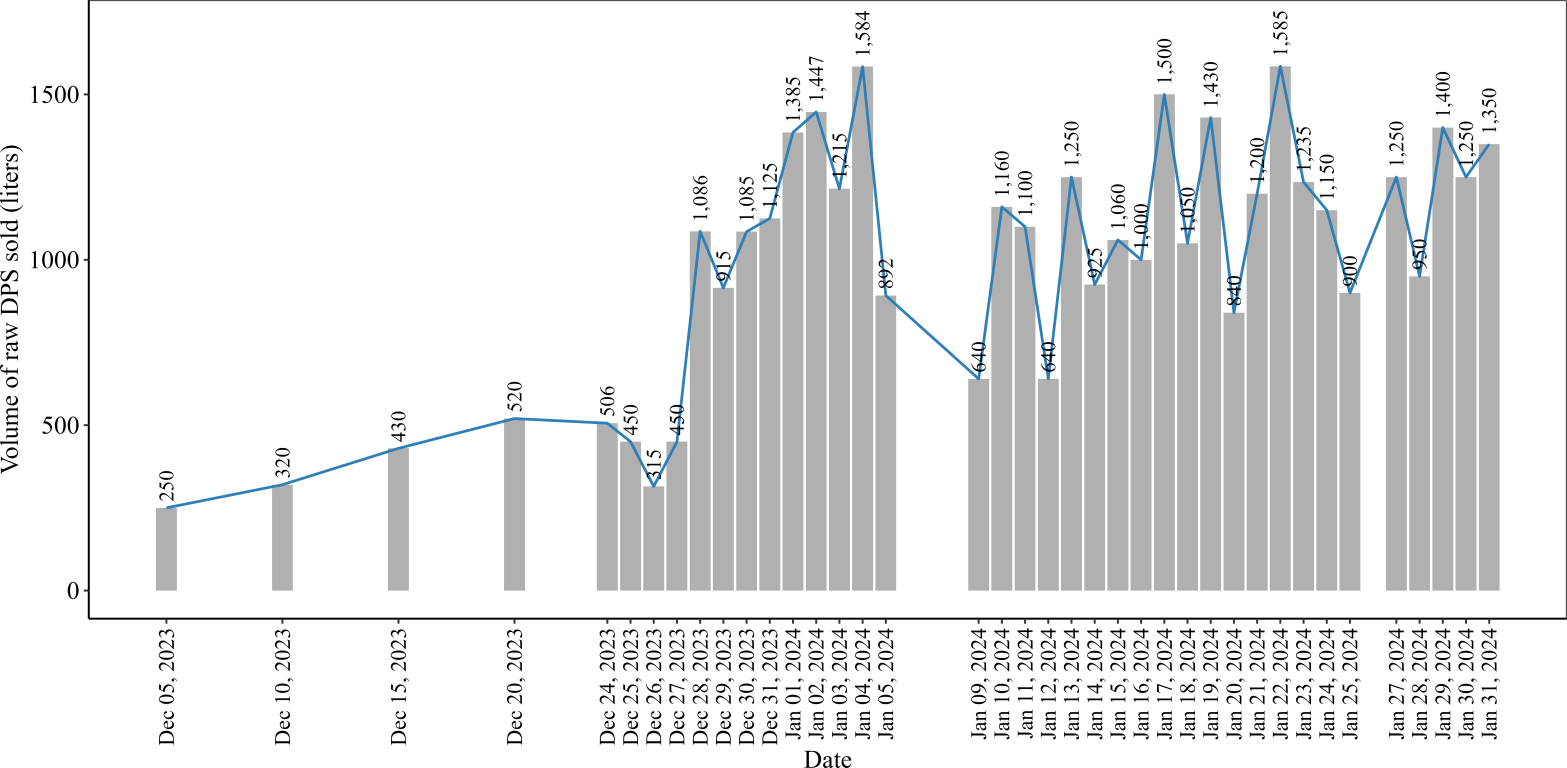
Daily sales pattern of raw date palm sap sold (DPS; in liters) from Rajshahi to other districts during the period from December 5, 2023, to January 31, 2024.

Figure 4 illustrates the daily sales of raw DPS (in liters) from a specific location in Rajshahi district to other districts. On average, 996 (SD 377) liters of raw DPS were sold on a daily basis. However, there was an increasing pattern in daily sales between December 5, 2023, and January 4, 2024, which started at 250 liters on December 5 and reached more than 6 times that amount by January 4, 2024. Subsequently, there was a fluctuating pattern in the daily sales of raw DPS, with an average of approximately 1120 (SD 251) liters.

**Figure 5.**
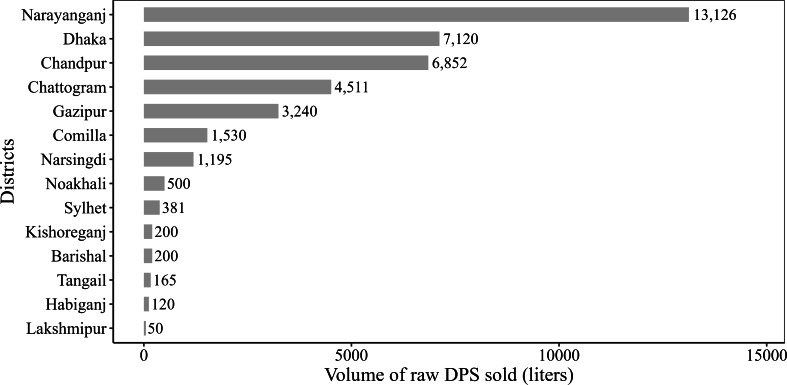
Quantity of raw date palm sap sold (in liters) from Rajshahi to other districts during the period from December 5, 2023, to January 31, 2024.

Figure 5 highlights the distribution of raw DPS, with quantities specified in liters, from a specific location in Rajshahi district to other districts during the period from December 5, 2023, to January 31, 2024. Our analysis revealed that raw DPS harvested in this particular place in Rajshahi district was sold to 14 different districts, with Narayanganj receiving the largest quantity (13,126/39,190, 33.5% liters) and Lakshmipur receiving the smallest (50/39,190, 0.1% liters). It was also revealed that the non–Nipah-prone areas (except Comilla and Tangail) received DPS from the Nipah-prone area (Rajshahi) of Bangladesh.

## Discussion

### Principal Findings

Raw DPS contaminated by bat excreta is considered the primary cause of the transmission of NiV in Bangladesh [4,8,10-12]. While this study cannot establish a direct causal relationship, understanding the role of digital marketing in distribution patterns is important for informing public health strategies. Hence, considering the impact of digital marketing of raw DPS on the risk of NiV transmission is imperative. In our study, we have found a strong correlation between digital marketing strategies and the expanded distribution of raw DPS across districts in Bangladesh. On the basis of observed data, digital marketing appears to be associated with wider dissemination of raw DPS; however, further research is needed to establish causality [5,17].

The observed peak in raw DPS–related content from mid-December to mid-January may be influenced by seasonal and cultural factors in Bangladesh. This period coincides with the winter harvest season for DPS, when cooler night temperatures favor sap flow and preservation, increasing both availability and demand. In addition, winter months are associated with seasonal foods, vacations in educational institutions, and informal social gatherings, which may further promote consumption and online sharing of raw DPS. While our study did not directly assess cultural events or weather variables, these contextual factors may partly explain the observed temporal patterns and warrant further investigation.

The marketing of raw DPS on digital platforms generated substantial online engagement, as shown by the high volume of interactions with raw DPS–related posts. Although these data cannot help identify the change in customer interest over time or compare it with a baseline level, the cross-sectional nature of the data limits temporal analysis of customer interest patterns. This is particularly concerning given the established correlation between raw DPS consumption and NiV transmission, highlighting that the public health agencies should establish digital infoveillance systems to monitor high-risk products and launch targeted risk communication campaigns on the same platforms [5,16,17].

Facebook reactions such as “wow,” “haha,” and “love,” as well as comments on the digital content, indicate user interaction with the content. While these engagement metrics indicate the interaction, they do not directly relate to the consumption behavior. The findings suggest a substantial level of human interaction with the posts related to raw DPS on social media, particularly on Facebook. Positive reactions such as “like,” “love,” and “wow” may reflect social approval or normalization of raw DPS consumption, while “wow” and “haha” reactions could indicate that the practice is perceived as novel, entertaining, or adventurous rather than risky. The positive engagement rate indicates increased interest in raw DPS, which may affect public perceptions and consumption behaviors. This pattern raises concern regarding the potential increase in the risk of NiV transmission, specifically in the absence of appropriate safety measures. This highlights the necessity of implementing as well as promoting safety interventions to reduce the risk of transmission [11,12,15].

The widespread distribution network through digital platforms signifies a ubiquitous availability and demand for raw DPS, which potentially increased the consumption of raw DPS across numerous districts in Bangladesh. In 2013, the first outbreak of NiV occurred in Dhaka district through the consumption of raw DPS brought from Mymensingh district [19]. This outbreak emphasizes the role of interdistrict distribution in contributing to the disease spread. In addition, a noticeable upward pattern was observed in daily sales of raw DPS in the Nipah-prone districts. This extensive distribution network, combined with an upward sales pattern of raw DPS, raises concerns about the potential transmission of NiV, as raw DPS is being sourced from districts belonging to Nipah-prone areas, particularly when considering the broad scope of distribution of raw DPS through digital marketing. The transmission risk cannot be quantified in this study. However, the identified patterns demonstrate the necessity of continuous monitoring and specify the public health intervention [10-12].

Our findings suggest that social media platforms, particularly Facebook and YouTube, could be leveraged for targeted digital public health interventions to reduce the risk of NiV transmission. Possible approaches constitute countermessaging to discourage the consumption of raw DPS and encourage safer options, including boiled sap, and conducting geo-targeted campaigns in districts receiving DPS shipments during the harvesting seasons. Platform-specific tools, such as sponsored posts and short video content delivered in locally appropriate languages, may enhance reach and engagement. Partnerships between public health authorities and social media platforms could extend support for timely risk communication and misinformation control.

### Limitations

This study has several limitations. First, a nonsystematic approach to collecting social media data may introduce selection bias and limit the representativeness of the relevant online content. Second, this study only summarized raw DPS promotional posts; however, it cannot establish any direct linkage between these online activities and specific NiV cases. Third, the on-site sales data came from only 1 vendor or location in 1 subdistrict; therefore, the sales volumes and patterns may not be representative of other vendors or Nipah-prone districts. Finally, laboratory testing was not conducted to establish whether the DPS under sale was contaminated with NiV or other pathogens, which limits our ability to evaluate the risk of actual infection. These limitations emphasize the importance of conducting more systematic future studies, including on-site observations in all Nipah-prone districts and laboratory testing of raw DPS that is intended to be sold.

### Conclusions

This study explores and documents how a known risk factor of NiV in Bangladesh—raw DPS—gains accelerated distribution across the country through social media promotion. Thus, the study elucidates the role of digital media and human behavior in the seemingly unpredictable spread of a disease, which has emerged as a novel way of transmission. Moreover, future research could explore how cultural aspects can increase (or decrease) the risk of disease transmission through modern media in other regions of the world. However, these phenomena will require rigorous analysis to be understood completely. Although social media platforms expedited the wide distribution and consumption of raw DPS in Bangladesh, these platforms facilitated the implementation of public health actions such as developing social media countermessaging campaigns, partnering with platform companies to flag high-risk posts, and delivering targeted education to digital marketers of raw DPS. The study’s conclusions support a multidisciplinary strategy that combines public health, policymaking, and digital media surveillance to handle the intricate problems brought on by the online marketing of goods such as raw DPS that are sensitive to health concerns.

## Abbreviations

DPS: date palm sap
icddr,b: International Centre for Diarrhoeal Disease Research, Bangladesh
NiV: Nipah virus
